# Severe gastrointestinal involvement in pediatric IgA vasculitis: a retrospective single-center cohort study in China

**DOI:** 10.3389/fped.2023.1194214

**Published:** 2023-08-08

**Authors:** Yifan Li, Xiaomei Zhang, Haimei Liu, Guomin Li, Wanzhen Guan, Tao Zhang, Qiaoqian Zeng, Yinv Gong, Hong Xu, Li Sun

**Affiliations:** ^1^ Department of Rheumatology, Children’s Hospital of Fudan University, National Center for Children’s Health, Shanghai, China; ^2^ Department of Nephrology, Children’s Hospital of Fudan University, National Center for Children’s Health, Shanghai, China

**Keywords:** IgA vasculitis, pediatric, severe, gastrointestinal involvement, treatment

## Abstract

**Objectives:**

The study aimed to describe the characteristics of gastrointestinal (GI) involvement in a cohort of hospitalized children with IgA vasculitis (IgAV) in China.

**Method:**

We reviewed the records of hospitalized IgAV patients from January 2014 to December 2020 at one tertiary medical center. The patients were divided into the severe GI group and the non-severe GI group according to the presence of massive GI bleeding and complications. The clinical manifestations, laboratory factors, and treatment were analyzed between the two groups.

**Results:**

A total of 1,179 patients were hospitalized due to IgAV. GI involvement was noted in 50% (589) of the patients, of whom 288 (48.9%) had severe GI involvement. GI complications were observed in 34 patients with IgAV with GI involvement. Rare onset age (<3 years or within 13–17 years), purpura above the waist, vomiting, high neutrophil-to-lymphocyte ratio, and decreased serum albumin were factors associated with severe GI involvement. Frequencies of renal involvement and biopsy-proven nephritis were higher in the severe GI group. The most commonly used medications were corticosteroids (100.0%) in the severe GI group. The maximum corticosteroid dose was higher (2.9 vs. 2.0 mg/kg), and more second-line therapies were needed (30.9% vs. 16.94%) in the severe GI group.

**Conclusions:**

Severe GI involvement in children is common in our center. Rare onset age, purpura above the waist, vomiting, high neutrophil-to-lymphocyte ratio, and decreased serum albumin are associated with severe GI involvement. Patients with severe GI involvement need higher doses of corticosteroids and second-line therapy.

## Introduction

IgA vasculitis (IgAV), known as Henoch–Schönlein purpura (HSP), is the most common systemic vasculitis in children, with an incidence of 10–20 per 100,000 (1). IgAV occurs most frequently between the ages of 3 and 12 years, and it is rare in children younger than 2 years old (2). IgAV is characterized by polymorphonuclear leukocyte inflammatory infiltration of small blood vessels and IgA1-predominant immune deposits (3).

The clinical manifestations of IgAV are non-thrombocytopenic purpura, arthritis or arthralgia, abdominal pain and gastrointestinal (GI) hemorrhage, and glomerulonephritis. Severe gastrointestinal involvement of IgAV includes massive gastrointestinal hemorrhage and abdominal complications, representing the primary cause of mortality in the acute phase of the disease. Unfortunately, the clinical manifestations of severe GI involvement always tend to be insidious and atypical (4). In previous studies, age, blood neutrophil-to-lymphocyte ratio (NLR), mean platelet volume (MPV), platelet-to-lymphocyte ratio (PLR), reduced activity of coagulation factor XIII, and clinically apparent edema were related to gastrointestinal involvement and gastrointestinal bleeding (5–9). There is currently no consensus therapy for treating severe IgAV with GI involvement. IgAV patients with severe GI involvement should be treated with a positive therapeutic strategy that includes routine supportive care, fasting, parenteral nutrition, high-dose corticosteroid (CS), and even surgical intervention (10, 11). As the most commonly used drug, corticosteroids at 1–2 mg/kg/day are the mainstay of therapy. In addition, effective use of pulse methylprednisolone, intravenous immunoglobulins (IVIG), blood purification, and immunosuppressive treatment, including cyclophosphamide (CYC) and mycophenolate mofetil, have also been reported in some case reports or retrospective case series (12–17).

Although some studies have reported the clinical manifestations, treatment, and prognosis of pediatric IgAV with GI involvement, the treatment and complications of different institutions are quite different. Furthermore, studies involving large pediatric cohorts of IgAV patients with GI involvement are limited. Therefore, our study aimed to describe the characteristics, management, and outcome of a cohort of hospitalized children with IgAV with GI involvement and to explore factors associated with severe IgAV with GI involvement in children.

## Materials and methods

### Study design and selection of patients

We retrospectively reviewed the medical records of patients under 18 years of age with a diagnosis of IgAV from January 2014 to December 2020 at one tertiary medical center in China. The criteria for hospital admission for IgAV in our center included severe joint pain or swelling, severe or intermittent abdominal pain, GI hemorrhage, evidence of nephritis/nephritic syndrome or renal impairment, neurological symptoms, and recurrent severe purpura. The diagnosis of IgAV was based on the validated criteria defined by the 2010 European League Against Rheumatism/Pediatric Rheumatology International Trials Organization/Pediatric Rheumatology European Society (EULAR/PRINTO/PRES) (18). Patients with underlying disease, chronic infectious disease, chronic renal disease, or inflammatory bowel disease were excluded from the study. GI involvement was defined as GI manifestations, including abdominal pain, nausea or vomiting, diarrhea, intestinal bleeding, and severe abdominal complications, after excluding other causes of GI symptoms. In the present study, patients hospitalized for GI involvement did not include patients who had GI manifestations before but were already discharged when hospitalized in our center.

We retrieved epidemiologic characteristics, clinical manifestations, laboratory data, radiological features, endoscopic features, organ involvement, medications, and other therapies of IgAV children with GI involvement. In addition, the families of the patients were interviewed by phone about renal involvement and relapse if they did not follow up in our center. Written informed consent was obtained from the subjects or their parents. The study was conducted in accordance with the principles outlined in the Declaration of Helsinki with approval from the Ethics Committee of the Children's Hospital of Fudan University [ethical number: (2021) 496].

The neutrophil-to-lymphocyte ratio, platelet counts, erythrocyte sedimentation rate (ESR), D-dimer, serum IgA level, albumin levels, urinalysis, and fecal occult blood test results were recorded at the time of diagnosis. Radiological tests and endoscopy, including abdominal ultrasonography and abdominal computed tomography, were also reviewed.

In our center, patients with obvious abdominal symptoms who failed supportive treatment received corticosteroids equal to a 2 mg/kg/day dose of prednisone. Once the symptoms did not improve, a massive dose of corticosteroid (2–10 mg/kg/day), pulsed intravenous methylprednisolone (10–30 mg/kg) with a maximum of 1 g/day for three consecutive days, and intravenous immunoglobulin (1–2 g/kg) were considered. Blood purification may be considered for patients still suffering from severe abdominal pain or massive GI bleeding after the treatment described above. In addition, second-line therapy was used for patients suffering from recurrent abdominal symptoms during CS reduction.

According to whether there was severe GI involvement, IgAV patients with GI involvement were divided into two groups: the severe GI involvement group and the non-severe GI involvement group. Severe GI involvement was defined as IgAV with massive GI bleeding (patients with positive occult blood were excluded) or severe abdominal complications, including intussusception, intestinal perforation, intestinal obstruction, pancreatitis, and hypovolemic shock. The clinical characteristics, laboratory features, and radiological features were analyzed between the two groups.

### Statistical analysis

All statistical analyses were performed using SPSS software ver. 23.0. Continuous data were defined as the mean and standard deviation (SD) and median (interquartile range), and the categorical variables were described as percentages. Visual (histogram and probability plots) and analytic (Kolmogorov–Smirnov/Shapiro–Wilk test) methods were used to determine whether the variables were normally distributed. We used the chi-squared test or Fisher's exact test to compare the categorical variables between the different groups and the Mann‒Whitney *U* test to compare differences between the two groups for continuous variables. The optimal cutoff value was determined through receiver operating characteristic (ROC) curve analysis, and then, each continuous parameter was converted into a classification variable. The optimal threshold in ROC curve analysis was chosen by maximizing Youden's index. Univariate and multivariate regression models were used to identify significant factors associated with severe GI involvement. A *p*-value < 0.05 was considered significant.

## Results

### Demographic and epidemiological data

A total of 1,179 patients diagnosed with IgAV were hospitalized in our center from January 2014 to December 2020. Among the 1,179 patients, the male-to-female ratio was 1.63:1 (731:448), and the median patient age at diagnosis was 5.7 (4.8–6.0) years, ranging from 1 to 17 years old. All patients fulfilled the EULAR/PRINTO/PRES classification criteria for IgAV (18). All patients (100%) presented with purpura, 534 (45.3%) patients had arthritis or arthralgia, 589 (50.0%) patients had GI involvement, and 574 (48.7%) patients had renal involvement. A total of 156 patients had GI involvement before but had already been released when they were hospitalized in our center. These patients were not included in the GI involvement cohort.

### Characteristics of IgAV with gastrointestinal involvement

Among the 589 patients with GI involvement, purpura was observed under the waist in 254 cases (42.80%) and above the waist in 335 cases (67.20%). Hemorrhagic bullous lesions developed in 26 (4.4%) patients. In addition, 192 (32.60%) patients had subcutaneous edema, mostly localized in the limbs, with 141 in the lower limbs and 46 in the upper limbs). In a minority of cases, angioedema was localized at the head (31 patients, 5.26%), scrotum (13 patients, 2.21%), and trunk (5 patients, 0.85%). All GI involvement patients had abdominal pain, followed by vomiting in 377 (64.01%) patients. GI bleeding was reported in 413 patients: 285 (48.39%) had massive GI bleeding, and 128 (21.73%) had positive occult blood stool tests. A total of 258 (43.80%) patients had hematochezia, and 89 (14.43%) patients had hematemesis. GI complications were observed in 34 (5.77%) patients, including intussusception (16 patients), intestinal obstruction (six patients), intestinal perforation (one patient), acute pancreatitis (six patients), and hypovolemic shock (eight patients). Among these patients with intussusception, 10 had small intestine intussusception, and the remaining six had ileum colon intussusception. Table 1 shows the clinical characteristics of the patients with GI involvement.

**Table 1:** Clinical characteristics of the severe and non-severe gastrointestinal involvement groups.

Characteristics	Severe GI involvement (*n* = 288)	Non-severe GI involvement (*n* = 301)		*p-*value
Total	288	301		
Onset age	5.5 (4.8–5.9)	5.9 (4.7–6.0)	*U* = 44,793.500	0.480
Onset age group			χ^2^ = 4.639	**0.043**
3–12	263 (91.3%)	288 (95.7%)		
<3 or >12	25 (8.7%)	13 (4.3%)		
Gender			χ^2^ = 0.855	0.394
Male	175 (60.8%)	194 (64.5%)		
Female	113 (39.2%)	107 (35.5%)		
Clinical manifestation				
Palpable purpura	288 (100%)	301 (100%)		
Hemorrhagic bullous lesions	13	13	χ^2^ = 0.013	0.908
Angioedema	98	94	χ^2^ = 0.525	0.483
Location of skin lesions			χ^2^ = 5.583	**0.020**
Lower extremities	110 (38.2%)	144 (47.8%)		
Above the waist	178 (61.8%)	157 (52.2%)		
Abdominal pain	288 (100%)	301 (100%)		
Vomiting	213 (74.0%)	164 (54.5%)	χ^2^ = 12.201	**0.001**
Massive GI bleeding	280 (97.2%)	0		
Hematemesis	89	0		
Hematochezia	258	0		
Fecal occult blood positive	285	128	χ^2^ = 223.709	**0.000**
Joint involvement	126 (43.75%)	141 (46.8%)	χ^2^ = 0.568	0.457
Renal involvement	121 (42.01%)	94 (31.23%)	χ^2^ = 7.385	**0.008**
Clinical type of renal involvement				
Hematuria	30	32		
Proteinuria	24	17		
Hematuria and proteinuria	67	45		
Biopsy-proven nephritis	27	12		
Ia	1	0		
IIa	8	3		
IIIa	12	6		
IIIb	6	3		
Complications	34 (11.80%)	0		
Intussusception	16	0		
Intestinal obstruction	6	0		
Intestinal perforation	1	0		
Acute pancreatitis	6	0		
Hypovolemic shock	8	0		

The *p*-values with statistically significant differences (*p* < 0.05) are highlighted in bold in the table.

### Comparison of clinical and laboratory features between the severe and non-severe GI involvement groups

According to the definition of severe GI involvement mentioned above, 288 (48.90%) patients were categorized into the severe GI involvement group, including 285 patients with massive GI bleeding and 34 patients with severe complications. Meanwhile, 301 patients were categorized into the non-severe GI involvement group. Demographic data, including onset age and sex, cutaneous manifestations, abdominal symptoms, laboratory investigations, and radiological and endoscopic features, were compared between the two groups. The onset age and sex showed no difference between the two groups. The patients were also categorized into two groups based on the age of onset of IgAV: patients with a rare onset age (<3 or >12 years) and those with a common onset age (3–12 years). More patients with a rare onset age were in the severe GI involvement group than in the non-severe GI involvement group (25 vs. 13, *p* = 0.043). Renal involvement was noted in 215 (36.5%) patients, and it occurred more frequently in the severe GI involvement group (121 vs. 94, *p* = 0.008), while joint involvement showed no difference between the two groups (Table 1).

Cutaneous manifestations, including the location of purpura, hemorrhagic bullous lesions, and angioedema, were compared between the two groups. Among these cutaneous manifestations, only the location of purpura showed a significant difference between the two groups. Purpura above the waist was more frequent in the severe GI involvement group (*p* = 0.02). A total of 11 patients in the severe GI involvement group had biliary stones, while none had biliary stones in the non-severe GI involvement group (Table 1). Vomiting was significantly more frequent in the severe GI involvement group among the abdominal symptoms (*p* = 0.001). With regard to laboratory features, the NLR, platelet (PLT) count, and D-dimer level were significantly higher in the severe GI involvement group than in the non-severe GI involvement group, with median values of 4.4, 368 × 10^9^/L, and 1.85 mg/L, respectively. However, the serum albumin (ALB) level and ESR were significantly lower in the severe GI involvement group. Serum IgA showed no difference between the two groups (Table 2).

**Table 2:** Laboratory features of the severe and non-severe gastrointestinal-involved IgAV children.

Laboratory features	Severe GI involvement (*n* = 288)	Non-severe GI involvement (*n* = 301)		*p*-value
Radiological features	275 (95.49%)	274 (91.03%)	χ^2^ = 4.617	**0.032**
CT scans	105 (36.46%)	56 (18.60%)		
Abdominal US	270 (93.75%)	266 (88.37%)		
Normal	150 (54.55%)	220 (%)	χ^2^ = 32.954	**0.000**
Thickening of intestinal wall	56	24		
Intestinal dilatation	35	6		
Biliary stone	27	4		
Pelvic fluid/ascites	49	19		
Endoscopic features				
Upper and/or lower GI endoscopies	120 (41.67%)	87 (28.90%)	χ^2^ = 10.518	**0.001**
Normal	0	10		
Ulceration	39	17		
Mucosal edema	8	1		
Mucosal ecchymosis/petechiae	85	61		
Erosion	19	11		
IgA (ELISA)	74	53		
IgA (+)	20	11		
Laboratory examination				
Neutrophil-to-lymphocyte ratio	4.40 (2.33–7.79)	2.86 (1.60–4.87)	*U* = 55,369.000	**0.000**
PLT (×10^9^/L)	368.00 (296.00–465.00)	342.00 (280.00–428.00)	*U* = 48,851.500	**0.008**
ESR (mm/h)	9.00 (5.00–18.50)	13.00 (7.00–23.50)	*U* = 38,030.000	**0.012**
ALB (g/L)	36.80 (32.58–41.20)	40.20 (37.50–42.90)	*U* = 30,190.000	**0.000**
IgA (g/L)	2.05 (1.54–2.69)	2.03 (1.57–2.70)	*U* = 42,981.500	0.971
D dimer (mg/L)	1.85 (0.95–5.42)	1.46 (0.79–3.54)	*U* = 49,187.500	**0.005**

The *p*-values with statistically significant differences (*p* < 0.05) are highlighted in bold in the table.

CT scans of the abdomen and/or abdominal ultrasound were performed in 549/589 (93.21%) patients, showing abnormalities in 179 (32.60%) patients, of whom 125 had severe GI involvement. Thickening of the bowel wall was the most frequent finding (80/179, 44.69%). Upper and/or lower GI endoscopies were performed in 207 patients, showing abnormalities in 197 patients, and the abnormalities were mostly mucosal ecchymosis or petechiae and mucosal ulcerations in 146 and 56 patients, respectively. The positive rate of IgA in biopsy tissue was only 24.41% (31/127) (Table 2).

### Rare onset age, vomiting, purpura above the waist, NLR, and serum albumin were associated with severe GI involvement

**Figure 1:**
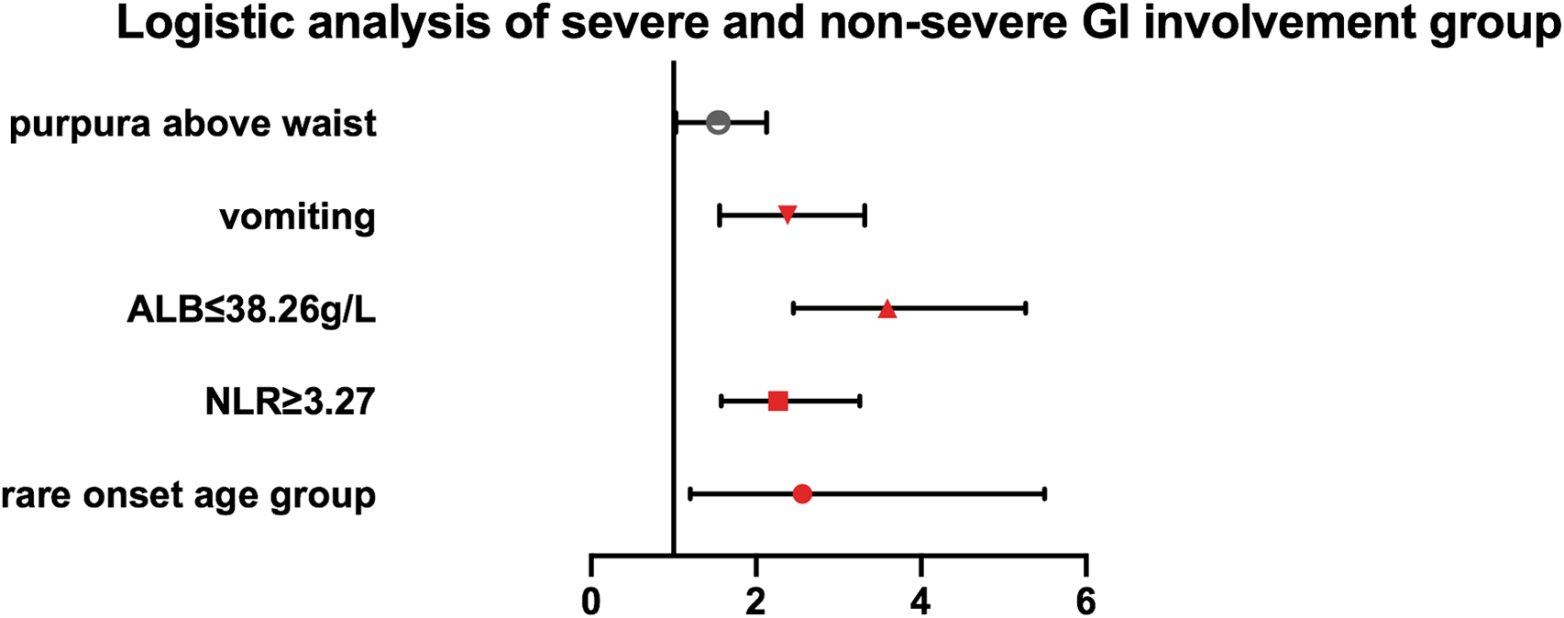
Multivariate logistic regression analysis of the association of rare onset age, purpura above the waist, vomiting, NLR, and serum albumin with severe GI involvement in IgAV patients.

According to the cutoff value, determined by receiver operating characteristic curve analysis, each continuous parameter was converted into a classification variable. Variables with *p* < 0.05 in the univariate analysis were included in the multivariable logistic regression analysis as the following covariates: rare onset age, vomiting, purpura above the waist, NLR (<3.27 vs. ≥3.27), PLT (<403.5 × 10^9^/L vs. ≥403.5 × 10^9^/L), D-dimer (<3.26 mg/L vs. ≥3.27 mg/L), and serum albumin (≤38.25 g/L vs. >38.25 g/L) (Supplementary Table S1). The results of the multivariable logistic regression analysis indicated that rare onset age, vomiting, location of skin lesion, NLR, and serum albumin levels were factors associated with severe GI involvement [rare onset age: odds ratio (OR) 2.561 (95% CI, 1.202–5.495; *p* = 0.015); vomiting: OR 2.273 (95% CI, 1.558–3.317; *p* = 0.000); purpura above the waist: OR 1.479 (95% CI, 1.029–2.125; *p* = 0.034); NLR ≥ 3.27: OR 2.267 (95% CI, 1.578–3.258; *p* = 0.000); and ALB ≤ 38.25 g/L: OR 3.592 (95% CI, 2.450–5.267; *p* = 0.000)] (Table 3, Figure 1).

**Table 3:** Multivariate logistic regression analysis for possible risk factors for severe gastrointestinal involvement in IgAV patients.

	*p*-value	OR (95% CI)
Rare onset age	**0** **.** **015**	2.561 (1.202–5.459)
Vomiting	**0**.**000**	2.273 (1.558–3.317)
Location of skin lesions	**0**.**034**	1.479 (1.029–2.125)
Neutrophil-to-lymphocyte ratio ≥ 3.27	**0**.**000**	2.267 (1.578–3.258)
Serum albumin ≤ 38.25(g/L)	**0**.**000**	3.592 (2.450–5.267)
D-dimer ≥ 3.26 (mg/L)	0.843	0.959 (0.637–1.445)

The *p*-values with statistically significant differences (*p* < 0.05) are highlighted in bold in the table.

### Treatment and outcome of IgAV patients with GI involvement

All patients received medical treatment, and 581 patients received immunosuppressive therapies, including 288 in the severe GI involvement group and 293 in the non-severe GI involvement group (*p* = 0.050). The most commonly used medications were CS, which were prescribed to 581/589 (98.64%) of the patients. A total of 441 (74.87%) IgAV patients with GI involvement received CS only, while 140 patients (23.77%) received second-line therapy, including methylprednisolone pulse therapy (pulse MP), IVIG, plasma exchange (TPE) or hemoperfusion (HP), and immunosuppressants. A total of 78 patients (13.43%) received methylprednisolone pulse therapy (15–30 mg/kg/day for 3 days, with a maximum of 1,000 mg/day), which was more frequently administered in the severe GI involvement group (56 vs. 22, *p* = 0.000). Eighty-three patients (14.1%) received intravenous immunoglobulin (0.5 g/kg/day for 2–4 days), including 58 patients in the severe group and 25 patients in the non-severe group (Table 4). Eight patients (1.4%) received plasma exchange or hemoperfusion, which was only used in the severe GI involvement group. Among these eight patients, five received pulse MP, IVIG, and CYC, and the other three received pulse MP and CYC before plasma exchange or hemoperfusion. CYC (71/581, 12.22%) was the most commonly prescribed immunosuppressant, followed by mycophenolate mofetil (5/581, 0.86%).

**Table 4:** Treatment of pediatric IgAV patients with gastrointestinal involvement.

	Severe GI involvement (*n* = 288)	Non-severe GI involvement (*n* = 301)		*p*-value
Immunosuppressive therapy	288 (100.00%)	293 (97.34%)		** **
CS only	199 (69.10%)	242 (80.40%)	χ^2^ = 14.465	**0.000**
CS ≥ 2 mg/kg/day	144	111		** **
CS 0.5–2 mg/kg/day	45	139	χ^2^ = 44.679	**0**.**000**
CS with other treatment	89 (30.90%)	51 (16.94%)	χ^2^ = 14.465	**0**.**000**
Pulse MP	56 (19.44%)	22 (7.31%)	χ^2^ = 17.803	**0**.**000**
IVIG	58 (20.14%)	25 (8.31%)	χ^2^ = 15.978	**0**.**000**
TPE/HP	8 (2.78%)	0 (0)		** **
Immunosuppressant	55 (19.10%)	21 (6.98%)	χ^2^ = 18.181	**0**.**000**
Cyclophosphamide	53	18		
Mycophenolate mofetil	2	3		
Maximum CS dose (mg/kg)	2.96 (2.00–4.70)	2.00 (1.60–3.00)	*U* = 58,572.000	**0.000**

CS, corticosteroids; Pulse MP, pulsed intravenous methylprednisolone; IVIG, intravenous immunoglobulin; TPE, plasma exchange; HP, hemoperfusion.

The *p*-values with statistically significant differences (*p* < 0.05) are highlighted in bold.

The medications and corticosteroids used significantly differed between the severe GI involvement group and the non-severe GI involvement group. All patients in the severe GI group used CS (100%). A total of 140 patients (56.0%) in the severe GI involvement group received doses of CS over 2 mg/kg, while 111/275 (36.8%) patients in the non-severe GI involvement group received doses of CS over 2 mg/kg (*p* = 0.000). The maximum dose of CS (mg/kg) in the severe GI involvement group was 2.90 (2.00–4.70) and was significantly higher than that in the non-severe GI involvement group [2.00 (1.60–3.00), *p* = 0.000]. One hundred ninety-nine patients in the severe GI group were successfully treated with CS only. However, 89 patients (30.9%) had to receive second-line therapy, which was significantly more than that in the non-severe GI group (16.94%) (*p* = 0.000). Table 4 shows the treatment of the severe and non-severe GI involvement groups.

Steroid treatment and immunosuppressants were successfully used in severe GI involvement patients, and all patients recovered from their abdominal symptoms. All patients survived without short bowel syndrome or colostomy, including the children with severe complications who required surgery. In the severe GI involvement group, 53/288 (18.40%) patients developed moderate to massive proteinuria or gross hematuria during follow-up, and 27 patients underwent a kidney biopsy. In the non-severe GI involvement group, 28/301 (9.30%) patients developed moderate to massive proteinuria or gross hematuria, and 12 patients underwent a kidney biopsy. So far, all patients have completely recovered, except for four patients from the severe GI involvement group who suffered proteinuria and hematuria.

## Discussion

To the best of our knowledge, this study is one of the large cohorts of pediatric IgA vasculitis with GI involvement in recent years. In our cohort, severe GI involvement accounted for 48.90% of the IgAV children with GI involvement, much higher than that of previous studies. The percentage of severe GI involvement in pediatric IgAV patients was 13.5%–17.6% in Taiwan (19), 63.10% in one study from Turkey (20), and 35% in a study from Italy (21). The high percentage of severe GI involvement may be related to the different admission standards of IgA vasculitis in different countries and regions. Abdomen pain was the most common symptom in our series, occurring in 100% (589/589) of the patients with GI involvement, and the second most common symptom was vomiting, similar to previous studies.

An abdominal ultrasound is considered the initial best diagnostic tool to assess GI involvement and complications in IgAV symptomatic patients (21). Abdominal ultrasound can help judge the severity of gastrointestinal inflammation. Consistently, 91.0% of the children in our cohort underwent abdominal ultrasound. When there is suspicion of abdominal complications, further radiological investigations can be performed, such as CT scans and GI endoscopy. Abdominal imaging mainly showed thickening of the intestinal bowel wall, while endoscopies showed more diverse lesions, including ulcerations, mucosal erythema, and/or purpura, as suggested in other series (22). While ultrasound has the advantages of being free of radiation and easy to use, its efficacy is contingent upon the skill level of the operator. If a patient experiences intense abdominal pain and abdominal ultrasound yields normal results, our institution prescribes a CT scan.

In our study, renal involvement was noted in 48.7% of pediatric patients with IgAV, similar to previous studies (23, 24). Renal involvement and biopsy-proven IgA nephritis were more frequent in the severe GI involvement group. However, severe GI involvement in the acute stage of IgAV imparts significant morbidity and mortality and may be associated with renal involvement (25). Our data showed that rare onset age, vomiting, purpura above the waist, NLR ≥ 3.27, and ALB ≤ 38.25 g/L were associated with severe GI involvement in pediatric IgAV patients, suggesting that these parameters may serve as possible biomarkers to predict or diagnose severe GI involvement in children with IgAV.

Among these clinical indicators with significant differences, the relationship between the onset age and the distribution of purpura and severe GI involvement of IgAV is worth noting. Several studies have demonstrated the relationship between onset age and manifestations of IgAV. Poterucha et al. (26) showed that adults under the age of 40 years had an increased risk of GI involvement. Liao et al. (19) reported that as onset age increased, pediatric patients were more likely to have renal involvement, and the risk of developing CS dependence and refractory disease increased with onset age. However, they found no significant difference in GI bleeding among their three onset age groups. In our study, children with an onset age of <3 years or within 13–17 years were more prone to severe GI involvement, which indicated that rare onset age might be a risk factor for severe GI involvement in IgAV children. In our study, the location of skin lesions and vomiting were related to severe GI involvement in IgAV children. Patients with purpura above the waist and vomiting were more likely to have severe GI involvement, which was seldom reported by previous studies. John et al. (27) reported that skin lesion distribution might predict long-term significant renal involvement in adult IgAV patients. However, further studies should be performed to determine the relationships.

In IgAV patients, proinflammatory cytokines such as interleukins and tumor necrosis factor are secreted in the acute phase because of inflammation (28). IgA anti-endothelial cell antibodies from IgAV patients activated endothelial cells to produce cytokines such as interleukin-8 and further resulted in inflammatory responses and neutrophil migration (29, 30). The association of the NLR with severe GI involvement in pediatric IgAV has been reported in many studies. The optimal cutoff value of the NLR for predicting GI bleeding was determined to be 3.90 (sensitivity of 87.5% and specificity of 88.6%) for adults and 2.86 (sensitivity of 73% and specificity of 68%), 2.82 (sensitivity of 81% and specificity of 76%), and 2.73 (sensitivity of 53.7 and specificity of 90%) for children (4, 31–33). The variation in cutoff values of NLR for severe GI involvement or GI bleeding may be due to the different sample sizes of these studies, the different ethnicities, and the different criteria for severe GI involvement. In the present study, the NLR was found to be associated with severe GI involvement in pediatric IgAV patients, and an NLR > 3.27 may serve as a biomarker to predict or diagnose severe GI involvement in children with IgAV.

Our study showed that hypoalbuminemia was associated with severe GI involvement in pediatric IgAV patients with a cutoff value of 38.25 g/L. Albumin was reported as a prognostic marker for ulcerative colitis (34). However, the cutoff value of 38.25 g/L is close to normal, and the clinical significance might be minimal. Further study is needed to identify the importance of albumin in patients with severe GI involvement.

Considering that the patients in our series were hospitalized with obvious GI involvement, all patients received medical treatment. Out of all the patients, 98.64% used oral or intravenous CS as first-line therapy. A massive dose of CS was more frequently used, and the maximum CS dosage was higher in the severe GI involvement group. More patients in the severe GI group needed second-line therapy, including methylprednisolone pulse therapy, intravenous immunoglobulin, intravenous cyclophosphamide, and mycophenolate mofetil. The most commonly used immunosuppression was cyclophosphamide. In our center, plasma exchange was used only for patients with severe GI involvement who failed to respond to the above treatment.

A multicenter, randomized study showed that the general use of IgAV can reduce GI symptoms and effectively alter the course of renal involvement (35). However, there is a lack of randomized research designed for IgAV patients with severe GI involvement with massive GI bleeding and GI complications. The manifestation of GI involvement in IgAV may exhibit different severities across different regions of the world, consequently leading to variations in treatment adopted by different medical centers. Rubino et al. (21) reported that steroids were used in 68% of hospitalized pediatric patients with GI involvement, and immunosuppressant drugs were used in only one patient. Liao et al. (19) showed that the most commonly used immunosuppressant medicine was azathioprine, followed by cyclosporine and hydroxychloroquine, which is quite different from our results.

The variation in the reported administration rate of CS and immunosuppression could be related to disease severity and practice differences in different studies. Since our patients had higher disease severity, they often required more aggressive treatment. The therapy varied in different medical constitutions. In our cohort, all secondary therapies seemed effective without severe side effects. Since CS is the mainstay of IgAV therapy, whether pulse MP is more effective than other second-line therapies is still unknown. Well-designed randomized controlled studies are needed to make evidence-based recommendations on treating IgAV patients with severe GI involvement.

There were several limitations in this cohort study. First, this was a retrospective study. A second limitation is the referral bias because our hospital is a tertiary referral hospital and one of the national centers for children's health in China. It is possible that our cohort may have a higher proportion of patients with severe illness, and it should be noted that our experience at a single center may not be representative of the overall population. Further confirmation of our findings by multicenter studies or nationwide databases is warranted.

In summary, the present study describes a large pediatric cohort of IgAV patients with GI involvement. GI involvement is common in IgAV, and the incidence rate of severe GI involvement was higher in our center. Rare onset age, vomiting, location of skin lesions, NLR, and serum albumin are associated with severe GI involvement. Patients with severe GI involvement need more high-dose corticosteroids and other immunosuppressive therapies, and their prognosis is generally good.

## Data Availability

The raw data supporting the conclusions of this article will be made available by the authors, without undue reservation.
